# Height at Late Adolescence and Incident Diabetes among Young Men

**DOI:** 10.1371/journal.pone.0136464

**Published:** 2015-08-25

**Authors:** Ariel Furer, Arnon Afek, Zivan Beer, Estela Derazne, Dorit Tzur, Orit Pinhas-Hamiel, Brian Reichman, Gilad Twig

**Affiliations:** 1 Department of Medicine I, Tel-Aviv Medical Center, Tel-Aviv, Israel; 2 The Israel Defense Forces Medical Corps, Tel Hashomer, Israel; 3 Israel Ministry of Health, Jerusalem, Israel; 4 The Sackler School of Medicine, Tel Aviv University, Tel Aviv, Israel; 5 Pediatric Endocrinology and Metabolism Unit, Edmond and Lily Safra Children’s Hospital, Sheba Medical Center, Tel Hashomer, Israel; 6 The Women and Children's Health Research Unit, Gertner Institute, Tel Hashomer, Israel; 7 Department of Medicine B, Sheba Medical Center, Tel Hashomer, Israel; 8 The Dr. Pinchas Bornstein Talpiot Medical Leadership Program, Sheba Medical Center, Tel Hashomer, Israel; Institute of Preventive Medicine, DENMARK

## Abstract

**Background:**

Short stature was suggested as a risk factor for diabetes onset among middle age individuals, but whether this is the case among young adults is unclear. Our goal was to assess the association between height and incident diabetes among young men.

**Methods and Findings:**

Incident diabetes was assessed among 32,055 men with no history of diabetes, from the prospectively followed young adults of the MELANY cohort. Height was measured at two time points; at adolescence (mean age 17.4±0.3 years) and grouped according to the US-CDC percentiles and at young adulthood (mean age 31.0±5.6 years). Cox proportional hazards models were applied. There were 702 new cases of diabetes during a mean follow-up of 6.3±4.3 years. There was a significant increase in the crude diabetes incidence rate with decreasing adolescent height percentile, from 4.23 cases/10^4^ person-years in the <10^th^ percentile group to 2.44 cases/10^4^ person-years in the 75^th^≤ percentile group. These results persisted when clinical and biochemical diabetes risk factors were included in multivariable models. Compared to the 75^th^≤ percentile group, height below the 10^th^ percentile was associated with a hazard ratio (HR) of 1.64 (95%CI 1.09–2.46, p = 0.017) for incident diabetes after adjustment for age, body mass index (BMI), fasting plasma glucose, HDL-cholesterol and triglyceride levels, white blood cells count, socioeconomic status, country of origin, family history of diabetes, sleep quality and physical activity. At age 30 years, each 1-cm decrement in adult height was associated with a 2.5% increase in diabetes adjusted risk (HR 1.025, 95%CI 1.01–1.04, p = 0.001).

**Conclusions:**

Shorter height at late adolescence or young adulthood was associated with an increased risk of incident diabetes among young men, independent of BMI and other diabetes risk factors.

## Introduction

The incidence rate of type 2 diabetes among children and young adults is increasing worldwide. Classic risk factors such as obesity or genetic variants account for only part of the increase in diabetes incidence [1], thereby emphasizing the need to explore additional risk factors to improve diabetes risk prediction.

Low stature was associated with abnormal fasting plasma glucose (FPG) [2] and diabetes in some studies [3–7], but not in all [8–11]. A recent meta-analysis concluded that height was associated with an increased diabetes risk in women, but not in men and indicated a high degree of heterogeneity among studies when controlling for diabetes risk factors [12]. The age of participants included in these studies is another source for ambiguity. Most studies included middle the characterized the height-diabetes association included middle aged participants spanning a range of over 3 decades [8,13,14], leaving paucity of data regarding young adults.

The Metabolic, Lifestyle and Nutrition Assessment in Young Adults (MELANY) cohort is a large ongoing, prospective study assessing risk factors for cardiovascular disease and diabetes [15–18] among young adults. This database also includes multiple measurements of height from late adolescence to young adulthood. Our goal was to assess the association between height at age 17 years and at young adulthood and incident diabetes among 32,055 young and apparently healthy men of this cohort.

## Research Design and Methods

### The MELANY cohort

The MELANY cohort is part of an ongoing investigation of the Israel Defense Forces (IDF) Medical Corps [19]. Israeli army personnel, older than 25-years of age, remaining in military service beyond the 2 to 3 years of mandatory service, are referred every 3 to 5 years for a routine health examination and screening tests at a screening center. At each visit the participants complete a detailed questionnaire assessing demographic, nutritional, lifestyle, and medical factors. Height and weight are measured, and a complete physical examination performed. Blood samples are drawn following a 14-hour fast and analyzed immediately. All medical information is recorded in the same central database, independent of scheduled visits, thereby facilitating ongoing, uniform follow-up as described previously [19]. All participants in the MELANY cohort, independent of their rank and position, have similar access to medical services which are provided free of charge [17]. In addition, prior to enlistment in the military at age 17 years, all MELANY participants underwent the mandatory, standard IDF pre-recruitment medical evaluation, comprising a physical examination including measurements of weight and height, assessment of cognitive performance and evaluation of socio-demographic data [17].

### Study population

**Fig 1:**
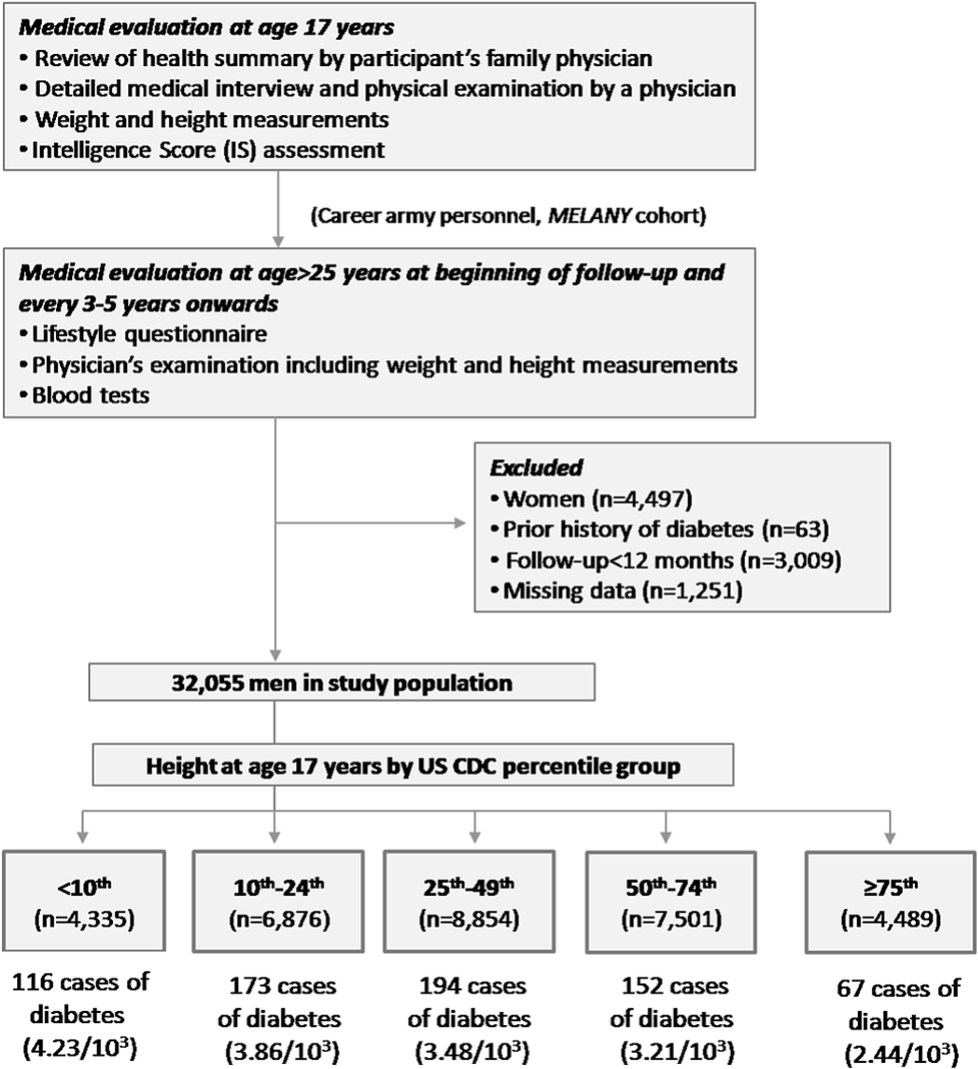
A Diagram of the study outcome and design.

Fig 1 shows a schematic diagram of the study design and its outcomes. Included in this study were men with complete measurements of weight and height at age 17 years, who attended the screening assessment at least once between January 1, 1995 and March 8, 2011. Since the first routine metabolic screen is performed at the first scheduled visit, participants who developed diabetes (type 1 or 2) prior to their first visit (n = 63) and those with a follow-up of less than 1 year from enrollment to the diagnosis of diabetes (n = 3,009) were excluded from the analysis (Fig 1). Participants with missing weight and height data at first visit were excluded from analysis (n = 1,251). The Institutional Review Board of the IDF Medical Corps approved this study without the need for participants' informed consent, given the assurance of strict maintenance of participants’ anonymity during data analyses. The MELANY dataset included 4,497 women, 28 of whom developed diabetes. This small number of incident cases precluded meaningful statistical analyses, and thus this study included only male subjects.

### Follow-up and outcomes

Follow-up began after the participants’ first visit to the screening center and ended at the time of diabetes diagnosis, death or retirement from military service or March 8, 2011, whichever came first. Screening for diabetes was performed at each visit to the center based on FPG levels. Incident cases of diabetes were based on a physician’s diagnosis of diabetes according to the American Diabetes Association criteria by documenting either two FPG levels ≥126 mg/dL (7.0 mmol/L) or a glucose level ≥200 mg/dL two hours after ingestion of 75 grams of glucose, conducted in cases in which the examining physician deemed the test necessary. All laboratory studies were performed on fresh samples, in an ISO-9002 quality-assured, core facility laboratory.

### Study variables

The Centers for Disease Control and Prevention (CDC) data were selected by the Israel Ministry of Health as the routine reference for anthropometric data for children, and have been reported to be appropriate for assessing Israeli children [20]. Accordingly, height and weight at adolescence were categorized according to the percentile cut-off points of the CDC for age (in months) and sex as previously reported [21]. Socio-economic status was based on place of residence and was obtained from records of the Israeli Ministry of Interior, which stratifies all municipalities according to 10 socio-economic decile groups determined by the Israeli Central Bureau of Statistics [17]. This scale considers age distribution, available workforce, level of unemployment, level of education (proportion of undergraduate students and those entitled to a high school diploma), average per capita income and proportion of income support recipients. Socioeconomic status was categorized into 3 groups: low (1^st^-4^th^ deciles), medium (5^th^ -7^th^) and high (8^th^ -10^th^) as reported previously [17]. Education was modeled as a categorical variable of low or high levels, with a threshold of 11 completed years of school education. This cut-off was chosen since it represents the maximum potential school instruction at the time of height measurement at adolescence. The intelligence score (IS), a Wechsler Adult Intelligence Score-equivalent IQ measure [22] and an independent risk for diabetes in this cohort [17], was treated as a continuous variable. Low intelligence scores reflect percent of scores lower than 100 points-equivalent in the Wechsler Adult Intelligence Scale. Country of origin, classified by the father’s or grandfather’s country of birth, was categorized into five geographical areas [17]: former USSR countries, Asia (non-USSR), Africa (excluding South Africa), Western (comprised of non-USSR Europe, North and South America, South Africa, Australia and New Zealand) and Israel. Country of birth was similarly classified. Data on mother's country of origin were not systematically obtained, and therefore were not included. Height at adulthood, body mass index (BMI), triglyceride and high-density lipoprotein cholesterol (HDL-c) level, FPG, and white blood cell (WBC) count at enrollment (first visit at the screening center) were treated as continuous variables. Smoking status (current smoker, past-smoker, never smoked), physical activity (≥150 min/week, <150 min/week, inactivity), breakfast consumption (frequent, sometimes, none), and family history of diabetes (yes, no) were treated as categorical variables. The mini sleep questionnaire (MSQ), a comprehensive index of sleep quality [23] and an independent risk factor for diabetes in this cohort [18] was treated as a continuous variable.

### Statistical analysis

Continuous variables were summarized using means and standard deviation (SD), or intra-quartile ranges (IQR) when variables did not exhibit normal distribution. Counts with percentages were used for categorical variables. The cohort was divided into 5 groups based on the CDC age-adjusted height percentiles at pre-recruitment evaluation [24]: <10^th^, 10–24^th^, 25–49^th^, 50–74^th^ and ≥75^th^ percentiles. P for trend were calculated using a linear regression where the independent variable was the 5 height categories and the dependent variable was the mean or proportion for each covariate (as shown in Table 1). ANOVA was performed to compare the mean of continuous variables among the study groups. Dunnet T3 and Bonferoni multiple comparison tests were used when homogeneity of variance test were rejected or not, respectively. Cox proportional hazard models were used to estimate the Hazard Ratios (HR) and 95% confidence intervals (CI) for developing diabetes among the 5 adolescent height groups and additionally, using height at enrollment as a continuous variable. The differences in follow-up among the 5 height groups were compared with ANOVA and Dunnet T3 post-hoc multiple comparison test. Several models were used to assess the diabetes-height relationships after adjusting for possible confounders such as age, sex, socioeconomic status and mediators such as FPG and physical activity as follows: Model 1, age at enrollment and birth year; Model 2, age at enrollment, birth year and BMI; Model 3, the variables of model 2 and metabolic risk factors (FPG, HDL-c and triglyceride levels and WBC count); Model 4, the variables of model 2 and lifestyle risk factors (physical activity, smoking status, sleep quality, breakfast consumption); Model 5, the variables of model 2 and socio-genetic risk factors (country of origin, family history of diabetes, IS, education, socioeconomic status). The final model (model 6) included covariates that were significantly (p<0.05) associated with diabetes risk in the age-adjusted model. As the effect of obesity status on the height-diabetes association was controlled by adjustment to BMI [9] or weight [25], we also analyzed the height-diabetes association using weight instead of BMI in the multivariate models (models 2b-6b at Table A in S1 File). The association between height at adulthood and incident diabetes was also assessed by dividing the cohort into 6 group with 5-cm increments (≤165 cm, 165< and ≤170 cm, 170< and ≤175 cm, 175< and ≤180 cm, 180< and ≤185 cm and ≥185 cm). The relationship between incidence rate of diabetes (per 1,000 person-years) and the median height of each group was fitted with linear and quadratic models.

**Table 1:** Characteristics of the study cohort at induction and enrollment to MELANY cohort by US CDC height percentile groups at age 17 years.

	Height at age 17 years (CDC-adjusted percentile groups)						
	<10^th^	10^th^-24^th^	25^th^-49^th^	50^th^-74^th^	≥75^th^	Total	p for trend
N	4,335	6,876	8,854	7,501	4,489	32,055	
**Characteristics at IDF induction assessment (age 17 years)**							
Age at assessment (years)	17.49±0.56	17.43±0.49	17.41±0.51	17.41±0.49	17.41±0.46	17.43±0.50	0.093
Height at age 17 (cm)	162.91±2.86	168.84±1.28	173.26±1.41	178.08±1.44	184.46±3.19	173.61±6.74	<0.001
Range (cm)	148–167	165–172	169–176	174–181	180–207	148–207	
BMI at age 17 (kg/m^2^)	21.2±3.1	21.3±3.0	21.4±3.0	21.4±3.0	21.6±3.2	21.4±3.0	0.005
Participants born in Israel (%)	78	82	83	85	86	83	0.011
Country of Origin (%)							
Israel	10	9	9	8	7	8	0.002
USSR	10	11	12	13	15	12	0.002
Asia	33	28	23	20	17	24	0.001
Africa	33	34	32	29	24	31	0.032
West	14	19	25	30	37	25	<0.001
Education ≥11 years (%)	85	89	92	94	96	92	0.002
Low intelligence score (%)	30.8	25.9	21.2	18.2	13.7	21.8	<0.001
Socioeconomic status (%)							
Low	38	35	33	30	26	32	<0.001
Intermediate	51	53	53	54	55	53	0.004
High	11	12	14	16	19	15	0.001
**Characteristics upon enrollment to MELANY cohort**							
Age (years)	32.32±6.2	31.46±5.89	30.96±5.63	30.48±5.31	29.69±4.81	30.96±5.64	0.001
Height at adulthood (cm)	167.50±4.08	172.46±2.99	176.47±2.77	180.84±2.75	186.86±3.83	176.87±6.62	<0.001
BMI (kg/m^2^)	25.07±3.95	25.37±3.90	25.46±3.96	25.56±3.98	25.64±4.02	25.44±3.96	0.012
Overweight (%)	35	38	37	39	38	38	0.040
Obese (%)	11	12	12	13	14	12	0.009
BP_Systolic_/BP_Diastolic_ (mmHg)	116.0±12.4/ 74.1±9.7	116.6±12.5/ 74.2±9.7	117.5±12.4/ 74.6±9.5	118.3±12.6/ 74.8±9.6	119.7±13.0/ 75.2±9.7	117.6±12.6/ 74.6±9.6	<0.001/ <0.001
FPG (mg/dL)	90.2±9.2	89.7±9.1	89.4±9.0	89.2±8.8	89.0±8.7	89.5±9.0	0.003
IFG (%)	13.9	12.9	11.7	11.2	10	11.9	<0.001
HDL-c (mg/dL)	46.7±11.0	46.5±10.7	46.2±10.7	45.9±10.5	46.0±10.7	46.2±10.7	0.026
LDL-c (mg/dL)	120.9±34.5	118.3±33.4	117.6±33.1	116.7±32.9	112.8±31.7	117.2±33.2	0.007
Triglycerides (mg/dL)[25^th^; 75^th^]	106[73;154]	101[71;149]	102[71;150]	100[72;145]	97[69;141]	101[71;148]	0.022
WBC count (1,000 cells/mm^3^)	6.77±1.58	6.69±1.49	6.69±1.50	6.61±1.47	6.56±1.41	6.66±1.49	0.004
Physical inactivity (%)	69	67	65	65	61	65	0.007
Abnormal MSQ score (%)	26.9	26.1	26.1	26.5	24.4	26.0	0.102
Family history of diabetes (%)	15.2	14.7	14.4	13.8	12.7	14.2	0.005
Past or current smokers (%)	44	44	41	38	36	41	0.001
Breakfast consumption (%)	83	82	81	80	76	81	0.007

For continuous variables, the mean (standard deviation) is given. MELANY, Metabolic, Lifestyle and Nutrition Assessment in Young Adults; IDF, Israel Defense Forces; BP, blood pressure; FPG, fasting plasma glucose; IFG, impaired fasting glucose; HDL-c, high-density lipoprotein cholesterol; LDL-c, low-density lipoprotein cholesterol; WBC, white blood cell; MSQ, mini sleep questionnaire.

Tests for linear trend across the height percentile groups at age 17 years and the diabetes risk estimates were assessed separately for each of the above-mentioned models using the midpoint of each height percentile group. Log minus log plots for each variable were inspected to verify the assumption of proportionality of the hazards. For all variables used in the model co-linearity was assessed using the Pearson correlation and the variance inflation factor (VIF). The maximal R recorded was 0.357 (triglyceride level and BMI at enrollment) with a maximal VIF of 1.34 (tolerance of 0.74) for triglyceride level. Subjects with missing data were excluded from the multivariable analyses (12.4% in model 6). Analyses were performed with SPSS statistical software, version 19.0 (SPSS, Inc., Chicago, IL, USA).

## Results

Table 1 shows the baseline characteristics of the cohort at age 17 years and upon enrollment into the MELANY cohort, according to the 5 height percentile groups at age 17 years. At the pre-recruitment (17 year old) assessment, subjects of short-stature (<10^th^ percentile) had BMI values lower by 0.4 kg/m^2^ than the high-stature group (≥75^th^ percentile; p = 0.001). Participants of Western origin predominate the tallest group (37%) as compared to pre-dominance of North African (33%) and Asian origin (33%) in the <10^th^ height percentile group. With respect to the tallest group, the <10^th^ height percentile group had a higher fraction of participants with lower intelligence (+17.1%), lower SES (+12%), immigrants (+6%) and low-level of education (+10%). At the first visit to the screening center the ≥75^th^ height percentile had higher fasting plasma glucose (+1.0 mg/dL; p = 0.001) and higher IFG rates (+3.9%) despite a higher BMI (+0.57 kg/m^2^; p<0.001) compared to the low stature group. The ≥75^th^ height percentile group had also lower rates of physical inactivity (61% vs. 67%), family history of diabetes (12.7% and 15.2%) and positive smoking history (-8%) as compared to the low-stature group. Of note, the height measured at young adulthood increased in all height percentile groups, compared to measurements in adolescence, with the largest increase (+4.59 cm) recorded in the <10^th^ height percentile group, compared with an increase of 2.40 cm in the ≥75^th^ height percentile group. Height at adolescence and adulthood were highly correlated (R = 0.92).

There were 702 new cases of diabetes diagnosed during 202,549 person-years of follow up. The incident rate of diabetes was 2.44/10^3^ person-years for participants in the ≥75^th^ height percentile group of height and increased with decreasing height percentile groups to 4.23/10^3^ person-years among those with height <10^th^ height percentile (Table 2). There was no difference in the length of follow-up among the study groups (p>0.25 in all comparison), with the exception of participants in the 10^th^-24^th^ percentile group who had longer follow-up; 6.51 years vs. 6.11–6.33 in the other study groups (p<0.021 compared to all other groups).

**Table 2:** Multivariable assessment of Hazard Ratios (HR) for developing diabetes by US CDC height percentile groups at age 17 years for different clusters of risk factors.

	Height at age 17 years (US CDC-adjusted percentile)					
	<10^th^	10^th^-24^th^	25^th^-49^th^	50^th^-74^th^	≥75^th^	Total or Mean or P for trend
N	4,335	6,876	8,854	7,501	4,489	32,055
New cases of diabetes	116	173	194	152	67	702
Mean age of diabetes onset (years)	38.67±6.92	37.99±6.84	37.26±6.79	36.80±6.70	35.81±6.53	37.30±6.81
Mean follow-up (years)	6.33±4.25	6.51±4.29	6.29±4.25	6.30±4.28	6.11±4.30	6.32±4.28
Person years of follow-up	27,421	44,760	55,658	47,290	27,419	202,549
Diabetes rate (per 1,000 person-years)	4.23	3.86	3.48	3.21	2.44	3.46
**Model 1: Age, birth year**						
HR	1.42	1.37	1.30	1.23	1 (Ref)	0.016
95%CI; P value	1.05–1.92;p = 0.023	1.03–1.82; p = 0.028	0.93–1.71;p = 0.065.	0.92–1.64; p = 0.156	-	
**Model 2: Age, birth year, BMI**						
HR	1.66	1.51	1.37	1.26	1 (Ref)	<0.001
95%CI; P value	1.28–2.25; p = 0.001	1.14–2.01; p = 0.004	1.03–1.81; p = 0.029	0.95–1.69; p = 0.11	-	
**Model 3: Age, birth year, BMI, FPG, HDL-c, triglycerides, WBC count**						
HR	1.67	1.52	1.37	1.25	1 (Ref)	<0.001
95%CI; P value	1.22–2.27; p = 0.001	1.14–2.04; p = 0.004	1.04–1.83; p = 0.027	0.93–1.68; p = 0.14	-	
**Model 4: Age, birth year, BMI, physical activity, smoking status, MSQ score, breakfast consumption**						
HR	1.76	1.70	1.45	1.18	1 (Ref)	<0.001
95%CI; P value	1.19–2.68; p = 0.005	1.18–2.44; p = 0.004	1.02–2.09; p = 0.040	0.82–1.73; p = 0.371	-	
**Model 5: Age, birth year, BMI, family history of diabetes, country of origin, intelligence score, socioeconomic status, education**						
HR	1.44	1.35	1.29	1.20	1 (Ref)	0.015
95%CI; P value	1.05–1.96;p = 0.023	1.01–1.80; p = 0.043	0.98–1.72; p = 0.075	0.89–1.61; p = 0.216	-	
**Model 6: Age, birth year, BMI, FPG, HDL-c, triglycerides, WBC count, socioeconomic status, country of origin, family history of diabetes, intelligence score, MSQ score, physical activity**						
HR	1.64	1.69	1.48	1.17	1 (Ref)	<0.001
95%CI; P value	1.09–2.46; p = 0.017	1.17–2.44; p = 0.005	1.03–2.12; p = 0.036	0.81–1.72;p = 0.39	-	

BMI, body mass index; FPG, fasting plasma glucose; HDL-c, high-density lipoprotein cholesterol; LDL-c, low-density lipoprotein cholesterol; WBC, white blood cell; MSQ, mini sleep questionnaire.

**Fig 2:**
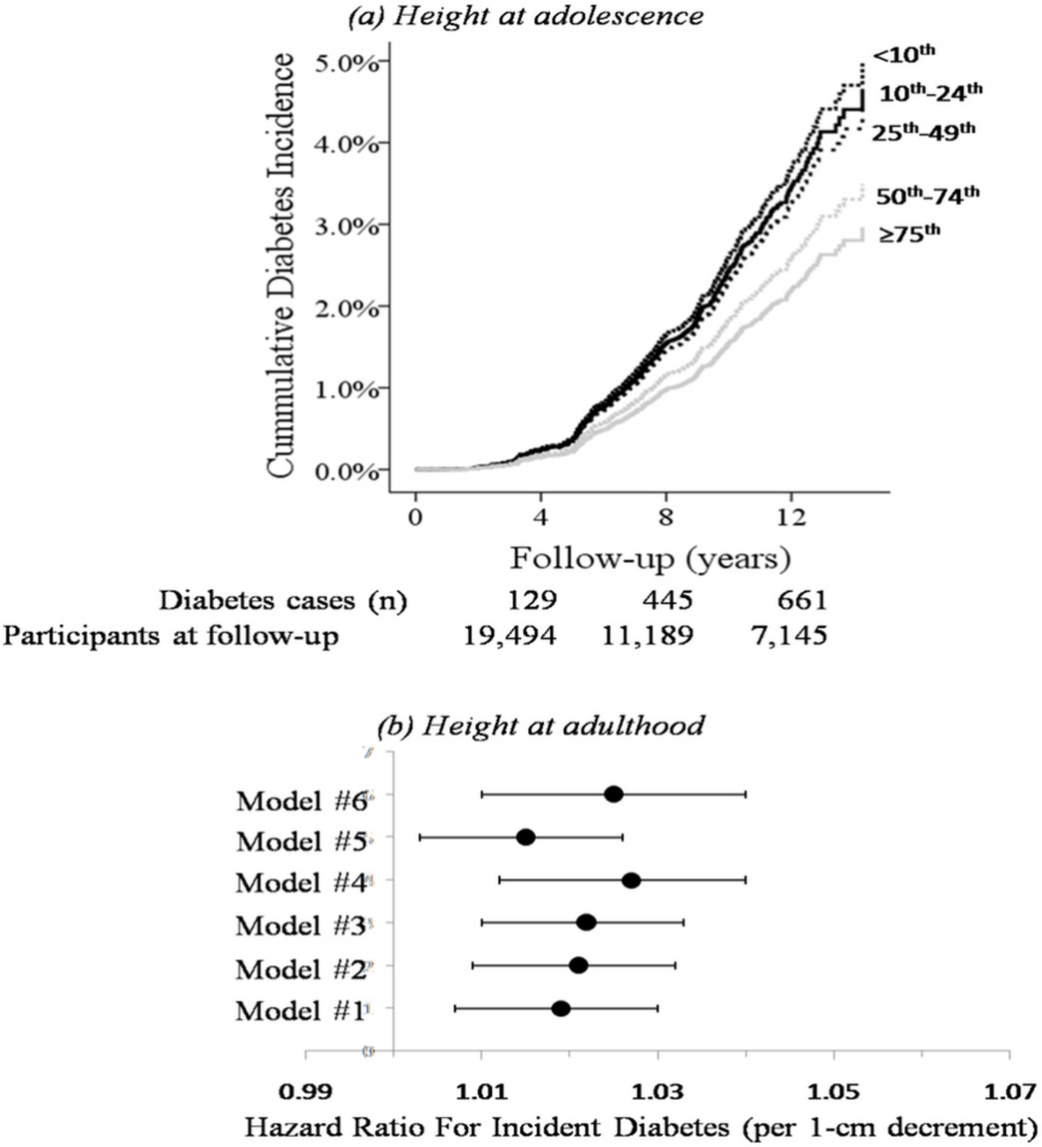
The association between height at adolescence and adulthood with incident diabetes. (a) Cox regression survival curves by US CDC height percentile groups were adjusted for age, birth year, BMI, FPG, HDL-c, triglycerides level, WBC count, socioeconomic status, country of origin, family history of diabetes, intelligence score, MSQ score and physical activity (model 6). Follow-up data and risk estimates are shown in Table 2. (b) The risk for diabetes incident is shown for each 1-cm decrement in height at adulthood (enrollment) for the different models used in the study (Table 2).

The independent effect of height percentile at age 17 years on the risk for incident diabetes persisted in the series of Cox models adjusted for socioeconomic, family history of diabetes, lifestyle and metabolic risk factors for incident diabetes as shown in Table 2. In the multivariable analysis adjusted for age, birth year, BMI, FPG, HDL-c, triglycerides level, WBC count, socioeconomic status, country of origin, family history of diabetes, IS, MSQ score and physical activity (model 6), participants in the lowest height group had 64% higher risk (HR 1.64, 95%CI 1.09–2.46, p = 0.017) for incident diabetes compared to the tallest group. Fig 2A depicts the Cox survival curves for cumulative diabetes incidence across the study groups after controlling for the variables in model 6. When a forward stepwise model was applied to the risk factors included in model 6, height at adolescence was included in the seventh step and was preceded by FPG, BMI, age, family history of diabetes, IS and MSQ sleep quality score.

**Fig 3:**
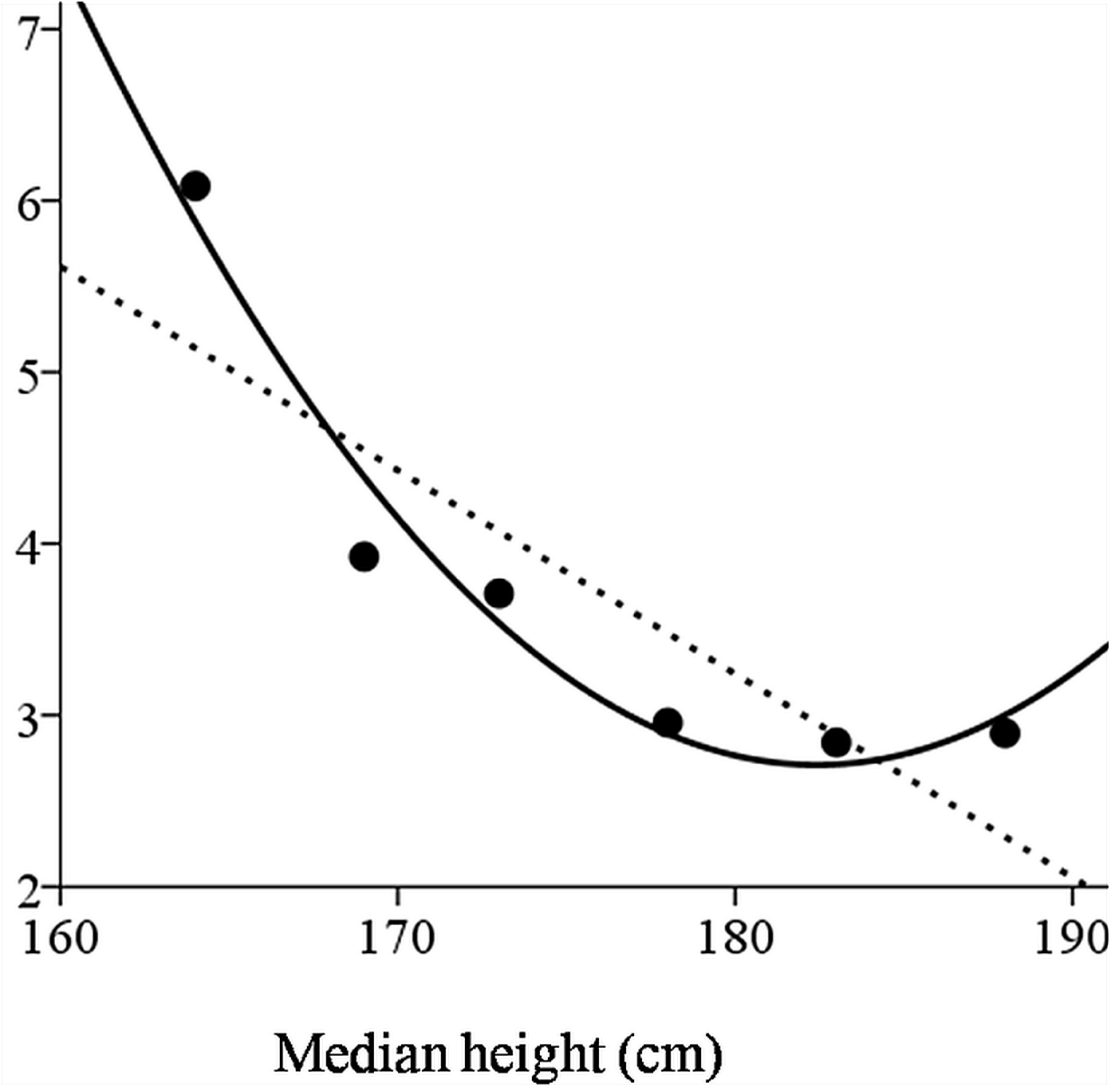
Diabetes incidence rate by height categories at adulthood. Data is shown based on the division shown in Table 3. A linear and quadratic fit had a goodness (R^2^) of 0.73 and 0.96, respectively.

The height measured at young adulthood (mean age 31.0±5.6 years) was also found to be independently associated with incident diabetes after controlling for the factors in models 1–6 (Fig 2B). In the multivariable analysis (model 6), the risk for incident diabetes increased by 2.5% for every 1-cm decrement in height (HR 1.025, 95%CI 1.01–1.04, p = 0.001) with similar results obtained in sensitivity analyses for the categories of socioeconomic status, country of origin or IS (Figure A in S1 File). We found similar results when height at adolescence was introduced to the model (model 6) for those measured between 17 and 18 years (n = 27,300; HR 1.024 95%CI = 1.008–1.041, p = 0.002). We also assessed the height-diabetes association using height at adulthood categories with 5 cm increments (Table 3). The incidence rate of diabetes decreased from 6.1 to 3.7 cases per 1,000 person-years in those shorter than 166 cm and those in the 171–175cm group, respectively, and plateaued for individuals taller than 175 cm. For clarity, this is shown in Fig 3. A linear and quadratic fit had a goodness (R^2^) of 0.73 and 0.96, respectively. These risk estimates persisted in multivariate analysis (model 6 inTable 2) with a significant increase for those shorter than 170 cm and a borderline significance for those in the 171–175cm group.

**Table 3:** The association between incidence diabetes and height categories at adulthood. Note that hazard ratio across categories were adjusted to model 6 in Table 2.

	Category of Height at adulthood					
Range (cm)	152–165	166–170	171–175	176–180	181–185	186–208
Median height (cm)	164	169	173	178	183	188
N	1,306	4,238	8,183	9,592	5,757	3,079
Incident diabetes cases	48	105	192	179	104	54
Follow-up (person years)	7,889	26,760	51,970	60,561	36,613	18,656
Incidence rate (per 1000 person years)	6.1	3.9	3.7	3.0	2.8	2.9
HR (95%CI)	2.00 (1.25–3.47), P = 0.001	1.52 (1.02–2.34), P = 0.038	1.42 (0.95–2.13), P = 0.08	1.22 (0.82–1.83), P = 0.32	1.07 (0.69–1.65), P = 0.76	1 (ref)

## Discussion

The current study of 32,055 men with 202,549 person-years of follow-up is to the best of our knowledge, the largest prospectively-designed cohort study assessing the relationship between height and incident diabetes. We demonstrated an inverse relationship between the risk for diabetes and height at both late adolescence and adulthood after adjustment for clinical and biochemical diabetes risk factors. The adjusted risk for incident diabetes increased by approximately 2.5% for every 1 cm decrement in height measured at adulthood (95%CI = 1.010–1.040, p = 0.001), with an adjusted HR of 1.64 (95%CI = 1.09–2.46, p = 0.017) among subjects of short stature (<10^th^ percentile) at adolescence compared to those in the upper height quartile. We found that the inverse relationship between height at adulthood and diabetes risk was not homogenous throughout the entire height range with a comparable risk for those taller than 175 cm and borderline risk for those at height 170–175cm. This finding is in agreement with Njostad et al [9] who also reported an inverse relationship between height and diabetes incidence with a cutoff of 168cm based on 87 cases. While most of the other studies, although not all [9], support a similar inverse association between height and incidence diabetes [3–5,7], the later was mostly examined among middle aged participants that spanned an age range higher than 3 decades [8,13,14].

There is a large heterogeneity in adjustment for confounders and mediators of diabetes. Socioeconomic status is associated with diabetes [26] and also with height [27,28], but was not controlled for in nearly half of the studies evaluating the relationship between height and diabetes [12]. In our study, the height-diabetes association persisted when three socioeconomic-related variables were controlled for (area of residence, education level, and cognitive performance). Limited access to healthcare services is another important factor that is associated with both low socioeconomic status and under-diagnosis of diabetes [29,30]. In this respect, the MELANY cohort is advantageous in minimizing these effects as all participants had similar and free of charge access to medical services with a scheduled screening program, independent of their rank and position.

Family history of diabetes was rarely adjusted in some of the studies assessing the relationship between height or hip circumference and diabetes [31,32]. In our study the height-diabetes association persisted after controlling for a family history of diabetes and participants' origin. It is noteworthy that the MELANY cohort comprises people from a wide range of different backgrounds and origins, since Israel is considered a “young” country with a relatively high rate of immigration. The young age of our participants at enrollment may limit the accuracy of reporting family history of diabetes, as it is possible that first degree relatives of participants ruling out a history of diabetes might develop diabetes in the future. For these participants, short stature may carry an additional risk to the risk associated with a positive family history of diabetes.

The participants' age at assessment is another source of ambiguity. The literature evaluating the height-diabetes association is predominantly based on studies including middle aged or older participants, or those with age ranges spanning 3 decades or more [6–8,13,14,25]. This age heterogeneity can bias the height-diabetes association either by the age-dependent decline in height that occurs in midlife onwards, or by an age-dependent susceptibility to obesity and metabolic abnormalities among young compared to older individuals [33], especially when metabolic variables are not controlled for [12]. In our study height was measured at two time intervals; late adolescence and young adulthood. The strict time interval of height measurement and adjustment for sex and age (by month) using US CDC height percentiles, together with a second measurement in young adulthood, minimizes the age-dependent bias.

Several childhood and metabolic mechanisms may underlie the association between height and diabetes. These include premature birth, irrespective of size for gestational age [34], fetal growth retardation especially when followed by a postnatal obesogenic environment [35], rapid weight gain in the first 3 months of life [36], low rates of linear growth during infancy [37], altered insulin-like growth factor-I levels [38], and vitamin D level and/or receptor polymorphism [39,40].

Our findings should be viewed in the context of the growing fraction of unexplained cases of diabetes. It is estimated that as many as half of the increase in the prevalence of diabetes cases occurring during the last three decades were not related to traditional risk factors [1,41]. The fact that the height-diabetes association persisted after adjustment for diabetes risk factors generally assessed in clinical settings, raises the possibility that height should be added to diabetes risk stratification among young men.

This study has several limitations. Firstly, our data does not include anthropometric measurements other than height, such as leg length, hip or waist circumference, and therefore could not assess the relative specificity of height compared to other anthropometric indices [4,42,43]. Secondly, no antibody data were available and as such the type of diabetes (eg, Type 1) could not be ascertained. However, we recently reported for this cohort that over 98% of diabetes-diagnosed cases were not prescribed insulin during the first year after diabetes diagnosis, thereby supporting type 2 predominance [17]. With that respect it is noteworthy that diabetes prevalence and age of onset was similar to other cohorts studying risk factors for type 2 diabetes of young adults [44–47].Finally, our study was limited to men. The strengths of the study include the large number of participants, standardized assessments and repeat measurements, rather than self-reported measurements [48] of weight and height, with adjustment for a large number of clinical, social, lifestyle and biochemical diabetes risk factors.

In conclusion, we found that height at adolescence or young adulthood was inversely associated with diabetes risk among young and apparently healthy young men after adjustment for clinical and biochemical diabetes risk factors. Our results suggest that height should be included in the diabetes risk stratification among young men.

## Supporting Information

S1 File Multivariable assessment of Hazard Ratios (HR) for developing diabetes by US CDC height percentile groups at age 17 years for different clusters of risk factors using weight rather than BMI (Table A). Sensitivity analysis for the effect of socioeconomic status (SES), country of birth and intelligence score on the height-diabetes association (**Figure A**).
(DOCX)
