# Correction: Height at Late Adolescence and Incident Diabetes among Young Men

**DOI:** 10.1371/journal.pone.0139183

**Published:** 2015-09-22

**Authors:** Ariel Furer, Arnon Afek, Zivan Beer, Estela Derazne, Dorit Tzur, Orit Pinhas-Hamiel, Brian Reichman, Gilad Twig

Fig 3 is incorrect. The label on the y-axis of Fig 3 is missing. The label should read ‘Incidence Diabetes Rate (per 1,000 person-years).’ The authors have provided a corrected version here.

**Fig 3:**
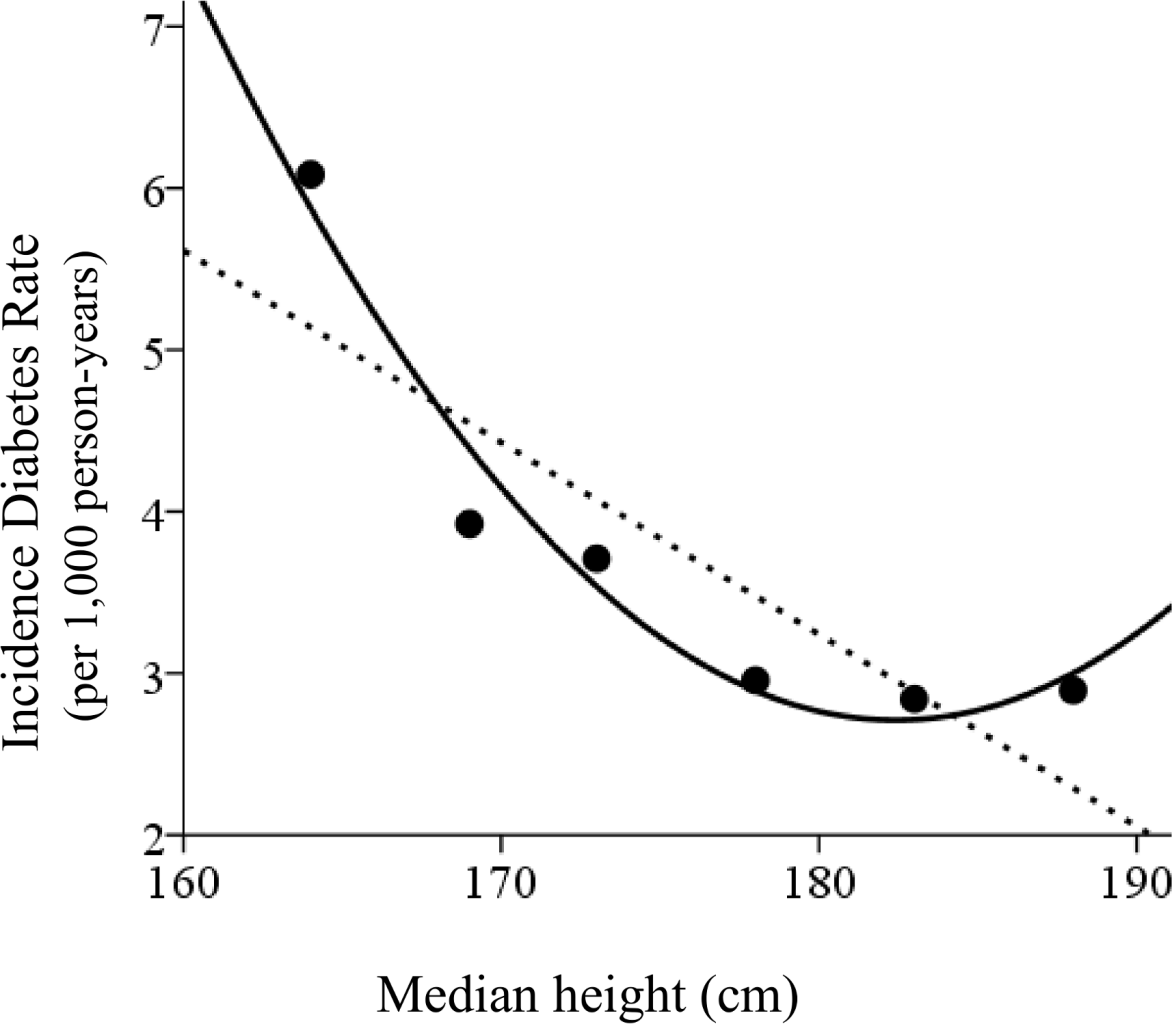
Diabetes incidence rate by height categories at adulthood. Data is shown based on the division shown in Table 3. A linear and quadratic fit had a goodness (R^2^) of 0.73 and 0.96, respectively.
