# Supplementary material for: Height at Late Adolescence and Incident Diabetes among Young Men
Source: PLoS One. 2015 Aug 25;10(8):e0136464. doi: 10.1371/journal.pone.0136464 (PMC4549289; doi:10.1371/journal.pone.0136464)
Supplement: S1 File — Sensitivity analysis for the effect of socioeconomic status (SES), country of birth and intelligence score on the height-diabetes association (Figure A). (DOCX) [file pone.0136464.s001.docx]

**Supporting Information Captions**

**File S1**

**Table A: Multivariable assessment of Hazard Ratios (HR) for developing diabetes by US CDC height percentile groups at age 17 years for different clusters of risk factors using weight rather than BMI.** Assessing diabetes risk by US CDC height percentile groups at age 17 years for different clusters of risk factors using weight rather than BMI. For comparison with adjustment that included BMI values, see Table 2.

|  | Height at age 17 years (US CDC-adjusted percentile) | | | | | |
| --- | --- | --- | --- | --- | --- | --- |
|  | **<10^th^** | **10^th^-24^th^** | **25^th^-49^th^** | **50^th^-74^th^** | ≥**75^th^** | **P for trend** |
| **Model 2b: Age, birth year**, **weight** | | | | | | |
| HR | 3.52 | 2.72 | 2.06 | 1.62 | 1 (Ref) | <0.001 |
| 95%CI; P value | 2.56-4.87;p<0.001 | 2.02-3.65;p<0.001 | 1.55-2.74;p<0.001 | 1.21-2.17;p=0.001 |  |  |
| **Model 3b: Age, birth year, weight, FPG, HDL-c, triglycerides, WBC count** | | | | | | |
| HR | 2.96 | 2.39 | 1.90 | 1.52 | 1 (Ref) | <0.001 |
| 95%CI; P value | 2.13-4.15;p<0.001 | 1.76-3.24; p<0.001 | 1.42-2.50; p<0.001 | 1.13-2.05; p<0.001 | - |  |
| **Model 4b: Age, birth year, weight, physical activity, smoking status, MSQ score, breakfast consumption** | | | | | | |
| HR | 3.61 | 2.97 | 2.18 | 1.51 | 1 (Ref) | <0.001 |
| 95%CI; P value | 2.37-5.52; p<0.001 | 2.04-4.35; p<0.001 | 1.51-3.15; p<0.001 | 1.04-2.21; p=0.032 | - |  |
| **Model 5b: Age, birth year, weight, family history of diabetes, country of origin, intelligence score, socioeconomic status, education** | | | | | | |
| HR | 2.95 | 2.36 | 1.93 | 1.51 | 1 (Ref) | <0.001 |
| 95%CI; P value | 2.13-4.11; p<0.001 | 1.74-3.19; p<0.001 | 1.45-2.57; p<0.001 | 1.13-2.02; p=0.006 | - |  |
| **Model 6b: Age, birth year, weight, FPG, HDL-c, triglycerides, WBC count, socioeconomic status, country of origin, family history of diabetes, intelligence score, MSQ score, physical activity** | | | | | | |
| HR | 2.51 | 2.42 | 1.94 | 1.39 | 1 (Ref) | <0.001 |
| 95%CI; P value | 1.60-3.92; p<0.001 | 1.63-3.60; p<0.001 | 1.33-2.84; p=0.001 | 0.94-2.05; p=0.095 |  |  |

**Figure A**: Sensitivity analysis for the effect of socioeconomic status (SES), country of birth and intelligence score on the height-diabetes association. In all analyses diabetes risk was adjusted to the variables in model 6 (see Table 2 for details). The p of interaction between height (CDC percentiles groups) and the given confounder is shown.

**
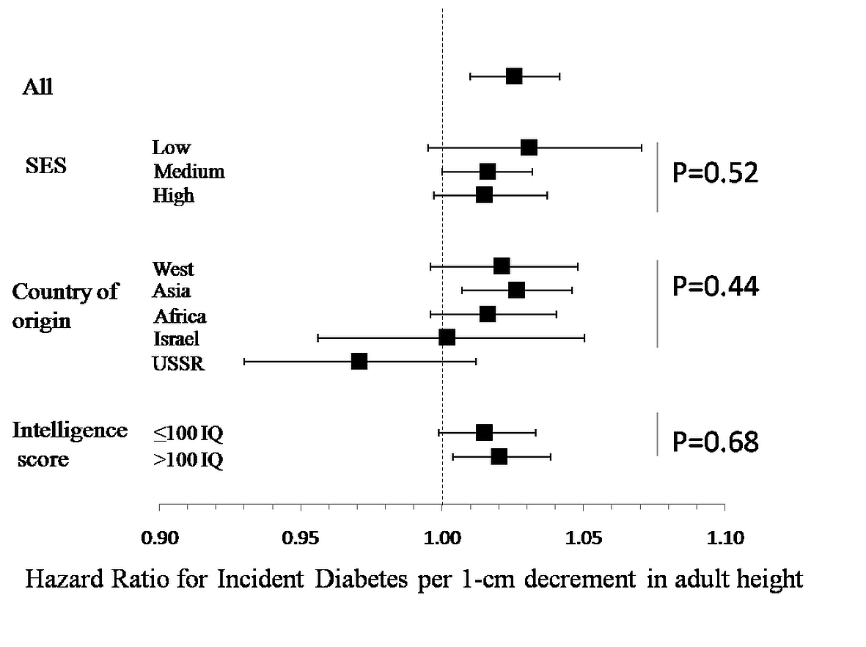
**
